# Letter from Dr. De Carro to Dr. Jenner

**Published:** 1803-05-01

**Authors:** J. De Carro

**Affiliations:** Vienna


### To Dr. JENNER,

DEAR SIR,

WHATEVER regards the progress of your discovery cannot be uninteresting to you; I remember the pleasure you expressed to me about four years ago, when I wrote to you that I had spread it beyond your island; I think myself bound in duty to procure to you nearly a similar pleasure by informing you of its arrival in the East India settlements, which has been effectuated by my means. You already know of its adoption at Constantinople; next to that famous town, I sent it to Bagdad, where it succeeded perfectly under the direction of Dr. Short, an English physician, attached to Mr. Harford Jones, British Resident at the court of His Highness the Pacha of Bagdad. From Bagdad it was sent to Bussora, where it likewise succeeded very well under the direction of Mr. Milne, an English surgeon, attached to Mr. Manetty, the British Consul. These four gentlemen have been exceedingly active in sending matter to several parts of Asia, and their correspondence with me on the subject contains a great number of interesting documents which will be a valuable addition to the history of your discovery.

From the first moment at which you made it known to the world, every ship bound to India was provided with vaccine matter, under every imaginable form; however, though innumerable trials were made by various practitioners, some of whom were acquainted with the practice, whilst in Europe, it never produced any effect. According to a very interesting account published in the Bombay Courier, July 3, 1802, by Drs. Moir and Scott, Members of the Medical Board at Bombay, and which has been transmitted to me in the original by the British Embassy at Constan-

Constantinople, the first vaccine pustule produced in the eastern world, (Bagdad and Bussora excepted) has been upon a black child, daughter of a servant of Capt. Hardie. Drs. Moir and Scott, describe in a lively manner the satisfaction which the whole town of Bombay expressed when they saw themselves in possession of that happy preventive; they speak of the encouragement which they experience from the government of that presidency, and mention some excellent measures they are taking to spread it rapidly into all the English settlements, even their hope of forwarding it to China. They are kind enough to make a very flattering mention of the efforts I have made to introduce the vaccination into the East, they take their principal instructions from my work on the subject, and they have taken much pains to explain to the Indoos and Parsees, who shew a great desire to have their children vaccinated, all the precautions necessary for acting with safety and certainty. The whole case of Count Mottet, so famous in the annals of the Cow-pox, has been well explained to the Asiatic practitioners into whose hands vaccine matter has been put. In short, the whole account is well written, and if you can get that newspaper, you will really find it worth your reading. How can one account for such a constant want of success with matter sent by sea, whilst that sent by land has succeeded immediately? Do you believe that the effluvia of tar, so strong in ships, or the sea air, has been the cause of it? It cannot be attributed to the heat of that latitude, for certainly it must be rather more intense at land. You know better than any body how delicate is the nature of vaccine matter. I have made some observations tending to prove that strong smells diminish its activity, particularly that of musk. Though I really regretted to see the vaccination adopted so late in the East Indies, I have felt a lively satisfaction in the idea of having been instrumental to its first introduction not only on the continent of Europe but on that of Asia. I shall besides observe, that it is a singular circumstance that the vaccine matter which I originally sent to Bagdad, and which has propagated itself so widely, was the product of the first experiment I made with the Cisalpine matter of Dr. Sacco; so you see that Great Britain has supplied the West, and Italy the East.

I congratulate you with all my heart for the decisive confirmation which your ingenious theory upon the origin of the Cow-pox has received from Dr. Loy's experiments. I found them so very important that I have given a French and German

German translation of his pamphlet, to which I have added some explanatory and introductory observations, and fragments of your correspondence, treating particularly on the subject. This little work has roused the attention of the continental physicians to the connection existing between the grease and the Cow-pox, and I do not doubt but what it will produce a great variety of new and interesting experiments. I do not remember whether I have already communicated to you an hypothesis which I began to form when I read your first work, but which seems to have acquired a certain degree of plausibility by Dr. Loy's experiments. I suppose that Asia being the cradle of the Small-pox as well as of Inoculation, it is not impossible that that scourge of mankind may have originated from that sort of cohabitation which many wandering nations of the East are said to have with their horses, which they have almost associated to their own existence. Could it not be possible that under some particular circumstances of climate, feeding, &c. the grease of those horses may have produced that variety which we know under the name of Small-pox. The connection between the grease, the cow-pox, and the small-pox cannot be denied; the grease must be the source of both, but where begins the variety, and what are the circumstances which favor its eruption, that is what merits some exact researches. We see, according to Dr. Loy, two sorts of grease, an eruptive and a local one. Has not perhaps the eruptive grease, communicated accidentally, been the cause of the variety *Small-pox*. How many diseases do we not know whose nature changes, which appear and disappear? Is not very often the same disease subject to varieties which have scarcely any resemblance? What a prodigious difference do we not perceive between a confluent Small-pox and the effects of inoculation; or even of natural infection producing but a few discrete pustules? Such changes may have taken place from the moment the original greasy virus was taken from the heels of horses. I consider this theory merely as an hypothesis, but I have amused myself to send a plan of researches to all physicians of the East with whom I correspond on the subject of Vaccination. The principal points of it are, to make enquiries whether the grease and the cow-pox are known in Asia? Whether any connection is supposed to exist among these two diseases of two different animals? Whether any mention of any of them is to be found in Asiatic, particularly veterinary physicians (if such exist)? In case the grease should be known in Asia, whether any documents cuments prove that it was known before the appearance of the Small-pox, or whether they are coeval? In short, I have suggested various such ideas which can at least give occasion to many researches, perhaps foreign to the subject I look for, but not less interesting. I even took occasion of a correspondence which I have begun, by means of the British Envoy here, with the Governor-general of the East Indies on the subject of Vaccination, to desire him to order his Medical Board to make every sort of research which can throw any light on that part of your theory, and to send me all the documents concerning it.

You may have perceived, by the contents of the xith. chapter of my work, second edition, that I rather inclined to consider the two diseases, the Cow-pox and the Small-pox, as a different species, or rather genus, without denying their analogy. You must, however, observe, that I rather paid attention to the external symptoms than to the nature of the disease itself, which I considered as too delicate a point to determine hitherto; but, as I said above, Dr. Loy's experiments have altered very much my opinion on the dissemblance of the two diseases. I have, besides, just now, a case which has made a strong impression upon me. I never saw as yet true vaccine pustules on any part of the body, but in a very few cases, where a sort of accidental vaccination could be supposed between the arm and the parts where they singly appeared. Last year, however, I saw a child attacked with what the French called *dartres*, and what you call, I believe, *tetters*; they were sore and much irritated when I vaccinated it; the pustules of the arms went on their regular course, but the sore spots of the elbow, shoulders, and back, were soon covered with large charateristic vaccine pustules, the fever was high, and ceased as soon as the black crusts began to form. Those crusts fell, and we had the pleasure to see the child's skin perfectly clean, free from the preceding eruption, and it has not appeared since. On last New-year's day I vaccinated the child of a physician, which had, for several months, a very abundant eruption of *crusta lactea* all over his face. As I had vaccinated several subjects under that eruption before, I did not hesitate to do the same. The vaccine pustule appeared regularly, but, on the 12th day, a most copious eruption of true vaccine pustules took place on the whole face, on a part of the back, where a blister had been kept open on account of the milk crust, and on tke scrotum, which happened to be slightly excoriated by the urine; the other parts of the body, where the skin was sound, sound, remained free from pustules. But, gradually, the face and the whole head swelled so much, that the eyes were closed during six days; I was very uneasy about them, and desired to call our excellent oculist, Dr. Beer, to examine them. He prescribed a dry fomentation with sacs of aromatic herbs and camphor, and, as collyrium, a weak solution of corrosive sublimate with some drops of laudanum. Luckily the eyes were opened on the sixth day, and were found perfectly sound. You may easily believe what a dreadful stroke it would have given to the vaccination, had an instance of blindness been put in its annals. I am very impatient to know whether the child will lose its former eruption on the falling of the vaccine black crust. The smell was precisely the same as that of the Small-pox; the appearance of the child's head was precisely that of a confluent Small-pox; the vaccine appearance, the depression in the centre, were, however, very distinct on the edges of the face, where some pustules were quite insulated. The mother and a very careful nurse are uncertain of their having had the Small-pox, but have stronger reason to suppose that they have not. Both of them have alternately carried the child day and night, its face resting on theirs. The mother has vaccinated herself on the temple and on the wrist, and the nurse on seven or eight places of her face; those pustules have actually the most regular appearance; I have taken matter from the mother's face and hand. This case is certainly very instructive; it shews the resemblance which the Cow-pox and the Small-pox can have under certain circumstances. I shall draw an important practical conclusion from it, viz. that no eruption should prevent vaccination, except when it is in the neighbourhood of the eyes.

The vaccination of sheep, in order to preserve them against the rot *(clavelée)* has engaged the attention of several gentlemen, proprietors of sheep farms. An experiment, on a very large scale, was instituted last summer in Hungaria; but, though it was made by the Professor of our Veterinary School, Dr. Pessina, a very learned and experienced veterinary physician, he never was able to produce on sheep or lambs any vaccine pustules. He had then recourse to the inoculation of the rot itself, and found there is the same proportion between the inoculated and the accidental rot as between the inoculated and the natural Small-pox. No sheep died, and they have all resisted it since the infection. I have myself lately made an excursion in Moravia with Baron Braun, proprietor of one one of the largest estates in this monarchy, in order to examine the state of his flocks attacked with the rot. The mortality was considerable, but, by a timely inoculation, (which I call in French *clavelisation, claveliser, &c.*) he has not lost any more. I never saw such a dreadful disease as the rot, it really gave me an idea of the plague; when it is of the bad kind, the sheep lose their eyes, their wool falls, the skin bursts and makes zig-zags, their nostrils are so much filled with a fetid matter, that the shepherds are obliged to syringe constantly with an infusion of elderberry flowers in order to prevent suffocation. The bad sort never produces distinct pustules from which matter can be taken for inoculation; it is only the benign sort, where they are distinct and contain fluid matter. Every pustule leaves a mark, and the wool never grows again; though I never saw that disease but once in my life, I was, as well as the oldest shepherds, able to say which sheep had already undergone the disease or not. The sick were separated from the healthy, just like cavalry regiments in a camp, the less sick from the incurable. In short, I saw plainly that the cure of the rot, or rather its prevention, must be a most important object of rural economy.

Our intention was to make some experiments upon the vaccination of sheep. We had brought a vaccinated child from Vienna, from which I took matter for six healthy sheep which I vaccinated myself. According to the journals of two very intelligent persons, whom we left there to examine the growth of the pustules, and to compare them daily with those of sixteen children which I vaccinated there, nothing was produced but local little sores. The sheep however were thrown amongst the sick, and two of them have, as could be expected, contracted the rot. Therefore little hope can be entertained of the vaccine being a preventive against it; but it appears to me sufficiently proved that *clavelisation* must be introduced in all sheep farms. I have, as you know, long ago proposed to make of the vaccination a sort of church regulation attending christening, but the clergy have been deaf to my proposal; as sheep are not christened, I hope that clavelisation will more easily be adopted, and that it will be the first operation they shall have to undergo after their birth. Has any thing been done in that respect in England?

I have taken the trouble of perusing a great number of books on the diseases of sheep, but I have found them upon the whole little instructive, and containing nothing more than empiric notions. I found, for instance, that many farmers farmers believe that the rot is not an original disease of sheep but the effect of the contagion of a certain eruptive disease to which hares are subject; they say that it is particularly frequent in farms where hares are in great quantity, and tame enough to play amongst the sheep. Have you any notion of that connection? enquire amongst your farmers. I would scarcely mention such a notion which is not supported by facts to any body else but to you; though I always was of opinion that popular traditions should not be blindly adopted, I always thought likewise that they should not be quite despised. The history of your discovery is such a striking proof of that principle that I venture to fix your attention upon the supposed connection between the eruption to which hares are subject and the rot of sheep. I believe besides that it would not be impossible to make experiments to determine whether the fact is grounded or not; could not one catch hares alive with nets, and inoculate to sheep any eruptive disease that should be observed on those animals?

Though I know very well how much your time is employed, can I flatter myself that you will answer this letter; I cannot have a greater pleasure than to receive one from you. You have a great many admirers, but surely none like me. God bless you.

*Vienna, Jan.* 22. 1803. J. DE CARRO, M.D.

P.S. I have introduced the vaccination at Salonica, and received from M. Lafont, a French physician, some very curious observations. He vaccinated in that town, where the plague has reigned almost constantly these six years, the son of the English Consul, who was the first upon whom he tried vaccination; he has been exposed to the plague in his father's house, where it carried off two servants; the child had symptoms which were declared by the most experienced people to whom the treatment of the plague is entrusted, (for you know that physicians are seldom called) to be those peculiar to that dreadful disease; a crisis took place, the child recovered. M. Lafont, who appears by his letters to be a well informed physician, is far from concluding that the Cow-pox is a preventive against the plague; but this case has roused his curiosity, and he proposes giving a particular attention to this circumstance. I do not remember where I have read that people who had undergone the Small-pox were less susceptible of catching the plague. I am curious to hear farther from M. Lafont, and if his correspondence affords any interesting observations,

I shall

I shall not neglect to communicate them to you. His first successful operation was performed with my ivory lancets. Not remembering whether I sent to you one, I include an impregnated one, from the pustule on the mother's face, accidentally vaccinated.

